# Analysis of expression profiles of MAGE-A antigens in oral squamous cell carcinoma cell lines

**DOI:** 10.1186/1746-160X-5-10

**Published:** 2009-04-09

**Authors:** Urs DA Müller-Richter, Albert Dowejko, Tobias Reuther, Johannes Kleinheinz, Torsten E Reichert, Oliver Driemel

**Affiliations:** 1 Dpt of Oral and Maxillofacial Plastic Surgery, University Hospital Würzburg, Pleicherwall 2, 97070 Würzburg, Germany; 2 Dpt of Oral and Maxillofacial Surgery, University Hospital Regensburg, Franz-Josef-Strauss-Allee 11, 93053 Regensburg, Germany; 3 Dpt of Maxillofacial Surgery, University of Münster, Waldeyerstraße 30, 48149 Münster, Germany

## Abstract

**Background:**

The immunological response to solid tumours is insufficient. Therefore, tumour specific antigens have been explored to facilitate the activation of the immune system. The cancer/testis antigen class of MAGE-A antigens is a possible target for vaccination. Their differential expression profiles also modulate the course of the cancer disease and its response to antineoplastic drugs.

**Methods:**

The expression profiles of MAGE-A2, -A3, -A4, -A6 and -A10 in five own oral squamous cell carcinoma cell lines were characterised by rt-PCR, qrt-PCR and immunocytochemistry with a global MAGE-A antibody (57B) and compared with those of an adult keratinocyte cell line (NHEK).

**Results:**

All tumour cell lines expressed MAGE-A antigens. The antigens were expressed in groups with different preferences. The predominant antigens expressed were MAGE-A2, -A3 and -A6. MAGE-A10 was not expressed in the cell lines tested. The MAGE-A gene products detected in the adult keratinocyte cell line NHEK were used as a reference.

**Conclusion:**

MAGE-A antigens are expressed in oral squamous cell carcinomas. The expression profiles measured facilitate distinct examinations in forthcoming studies on responses to antineoplastic drugs or radiation therapy. MAGE-A antigens are still an interesting aim for immunotherapy.

## Background

Tumour cells express specific antigens. Despite the fact that the protein products of these genes are absent or only partially found on healthy cells, the immunological response is insufficient[1,2]. The goal of several studies was to map these tumour antigens and use them to induce or boost the immunological response[3-5]. Of particular interest are tumour antigens that occur only on tumour cells and are not detectable on physiologically healthy cells. Such a group of tumour antigens are the MAGE-A antigens, a subgroup of cancer/testis antigens. These antigens are only expressed on germ cells and placenta cells[6,7]. The authors could also demonstrate an expression in fetal oral keratinocytes[25] but could not elucidate their role in development. In contrast, these antigens are commonly expressed on many different tumours[7-9]. They are found in dermal and oral squamous cell carcinomas, amongst others[7,10-12]. These studies suggest that MAGE-A antigens are simultaneously expressed in antigen groups. The MAGE-A subgroups differ in their protein structures[7,13]. This might influence an interaction with a potential drug or antibody and weaken their therapeutic effect. To validate this hypothesis, we quantitatively analysed the expression profiles of MAGE-A2, MAGE-A3, MAGE-A4, MAGE-A6 and MAGE-A10 in 5 oral squamous cell carcinoma cell lines by qrt-PCR and compared the results with the expression profile of a reference adult keratinocyte cell line.

## Methods

### Normal Human Epidermal Keratinocytes (NHEK)

The adult Normal Human Epidermal Keratinocytes (NHEK-adult) cell line was obtained from PromoCell GmbH, 69126 Heidelberg, Germany. The cell line was established using adult keratinocytes. The culturing was carried out according to the manufacturer's instructions.

### Tumour cell lines

#### PCI-13-1

The PCI-13-1 cell line was established from a male patient who suffered from low grade oral squamous cell carcinoma of the retromolar triangle. The tumour stage was pT4pN1M0G3.

#### PCI-1-1

The origin of this cell line was a larynx carcinoma of the glottis. It was harvested from a male patient. The grading was moderately differentiated and the tumour staging was pT2N0M0G2.

#### PCI-52

This tumour originated from the aryepiglottic fold of a male patient. It was a primary carcinoma with moderate differentiation. The tumour staging at the time of harvesting was pT2N0M0G2.

#### PCI-68-1

This cell line was established from a primary tongue carcinoma of male patient. The carcinoma was well differentiated. The tumour staging at time the cells were harvested was pT4N0M0G1.

#### PCI-9-1

This cell line was established from a primary carcinoma of the base of the tongue of a male patient. It was moderately differentiated. The tumour staging was pT4N3M0G2.

### RNA-isolation and rt-PCR

Total RNA from the tumour cell lines examined was extracted using RNeasy Mini Kits (Qiagen, 40724 Hilden, Germany) according to the manufacturer's instructions. The isolated RNA was stored at -20°C until reverse transcription. CDNA was created from isolated total RNA using dN6-random-primers (Roche Pharma AG, 79639 Grenzach-Wyhlen, Germany) and reverse transcription with *Superscript II *(Invitrogen GmbH, 76131 Karlsruhe, Germany). cDNA was incubated with 1 μl RNase A (Roche Pharma AG, 79639 Grenzach-Wyhlen, Germany) for 60 min at 37°C. The cDNA was stored at -20°C until rt-PCR analysis. RNA integrity was tested by rt-PCR of the housekeeping gene beta-actin. Specific rt-PCR detection of MAGE-A2, MAGE-A3, MAGE-A4, MAGE-A6 and MAGE-A10 was performed with the primers listed in Table 1. The primers were obtained from TibMolBiol (12103 Berlin, Germany). The ideal annealing temperature of single MAGE-A primers was defined by a gradient rt-PCR (52 to 72°C in 12 steps). The following program was used for MAGE-A primers: initial denaturation at 94°C for 5 minutes, 35 cycles of amplification with denaturation at 94°C for one minute, primer annealing for 1 minute (for specific temperatures see Table 1) and elongation at 72°C for two minutes, and a final elongation at 72°C for 10 minutes. The rt-PCR program for MAGE-A4 differed in that it used an elongation time of two minutes within the cycles and a final elongation time of 5 minutes. The synthesised rt-PCR products were separated by electrophoresis in an agarose gel, stained with ethidium bromide and visualised with UV light. As indicator for product size the 100 bp marker TrackIt kit (Invitrogen GmbH, 76131 Karlsruhe, Germany) was used. Water instead of cDNA was used as control.

**Table 1:** This table presents the structure of the primers used, their base pair lengths and the corresponding annealing temperatures.

**Gene**	**Sequence (5'→ 3')**	**Base pairs (bp)**	**Annealing temp. (°C)**
**β-Actin**	for CTACGTCGCCCTGGACTTCGAGC rev GATGGAGCCGCCGATCCACACGG	384	68

**MAGE-A2**	for AAGTAGGACCCGAGGCACTG rev GAAGAGGAAGAAGCGGTCTG	236	62

**MAGE-A3**	for CCAAGCAGCACTCAGTAGGAAGG rev GGAAGCTTTGCTGAAGATC	150	51

**MAGE-A4**	for GAGCAGACAGGCCAACCG rev AAGGACTCTGCGTCAGGC	445	65

**MAGE-A6**	for GGAAGGTGGCCAAGTTGGTTC rev CCAGCTGCAAGGAATCGGAAG	149	56

**MAGE-A10**	for CACAGAGCAGCACTGAAGGAG rev CTGGGTAAAGACTCACTGTCTGG	484	60

### Quantitative Real-Time PCR (LightCycler)

The expression profiles of MAGE-A2, MAGE-A3, MAGE-A4, MAGE-A6 and MAGE-A10 were quantitatively measured by qrt-PCR. To validate the values measured for each MAGE-A gene, three measurements were performed and the mean value calculated. The measurements were performed with a LightCycler 2.0 qrt-PCR-System (Roche Pharma AG, 79639 Grenzach-Wyhlen, Germany) using FastStart DNA Master Plus SYBR-Green I (Roche Pharma AG, 79639 Grenzach-Wyhlen, Germany). The reaction volume of each measurement was 20 μl, consisting of 1 μl cDNA, 1 μl forward primer (20 μM), 1 μl reverse primer (20 μM), 4 μl LightCycler DNA Master SYBR-Green I (Roche), and 13 μl water. The steps for the qrt-PCR program for the LightCycler were: an initial denaturation at 95°C for 7 minutes, 45 cycles of amplification with denaturation at 95°C for 10 minutes, primer annealing for 10 seconds (for specific temperatures see Table 1) and elongation at 72°C for 18 seconds. Completing the protocol, a melting range analysis with one cycle at 95°C for 30 seconds followed by a cycle at 67°C for 20 seconds with continuously measured fluorescence was performed. The values measured were normalised to the expression in adult keratinocytes (NHEK, reference)[25]. The values are given in arbitrary units (a.u.).

### Immunocytochemistry

For immunocytochemistry, the monoclonal global MAGE-A antibody 57B was used (by courtesy of Prof. Giulio C. Spagnoli, Onkologische Chirurgie, Institute for Surgical Research and Hospital Management, University Hospital Basel, 4031 Basel, Switzerland). This monoclonal antibody binds to a common epitope of MAGE-A antigens and facilitates simultaneous detection. The cells were seeded (5 × 10^5 ^adherent cells per chamber) on a four chamber slide (LabTek™ Chamber Slide System, Nunc, 65201 Wiesbaden, Germany) and incubated. Afterwards the cells were washed three times (Wash buffer S3006, DAKO, 22083 Hamburg, Germany) and fixated with acetone. Endogenous peroxidase was blocked with for ten minutes (DAKO 2023, DAKO, 22083 Hamburg, Germany) and the cells were washed again three times. The antibody ligands were blocked with 5% goat serum (X0907, DAKO, 22083 Hamburg, Germany) for one hour. The monoclonal antibody (anti-MAGE 57B) was diluted 1:10 and incubated with the fixed cells for one hour. The cells were washed three times again. Staining was performed with a secondary antibody (radish peroxidase) with the Envision™/HRP-System (DAKO K4063, DAKO, 22083 Hamburg, Germany) according to the manufacturer's instructions and additional counterstaining was done with hematoxylin.

## Results

**Figure 1:**
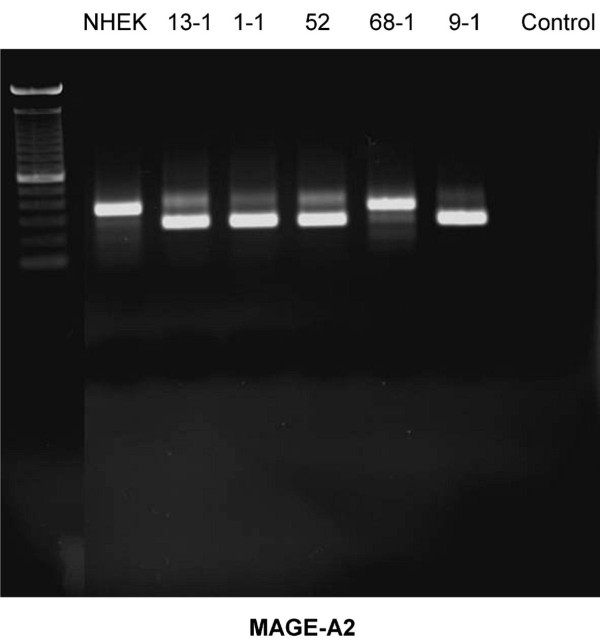
**RT-PCR-Blot of MAGE-A2 expression in the cell lines examined**. Double bands indicate splice variants. (left column = 100 bp marker, right column (control) = water).

**Figure 2:**
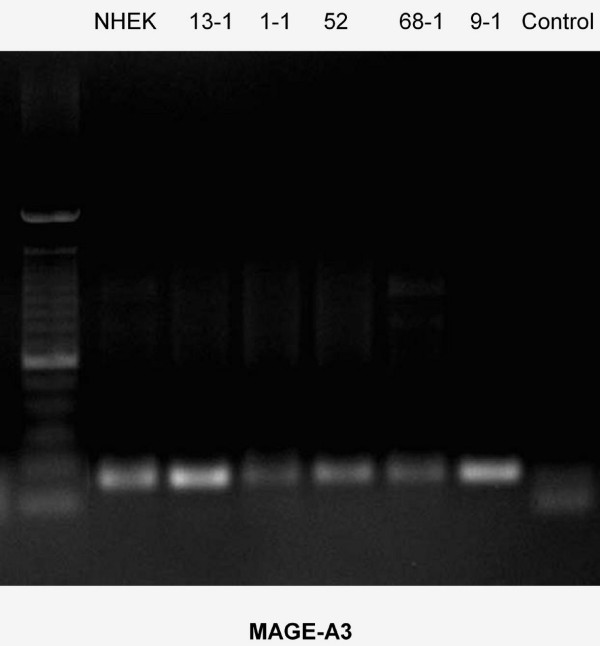
**RT-PCR-Blot of MAGE-A3 expression in the cell lines examined**. The intensity of the bands suggests different levels of expression. (left column = 100 bp marker, right column (control) = water).

**Figure 3:**
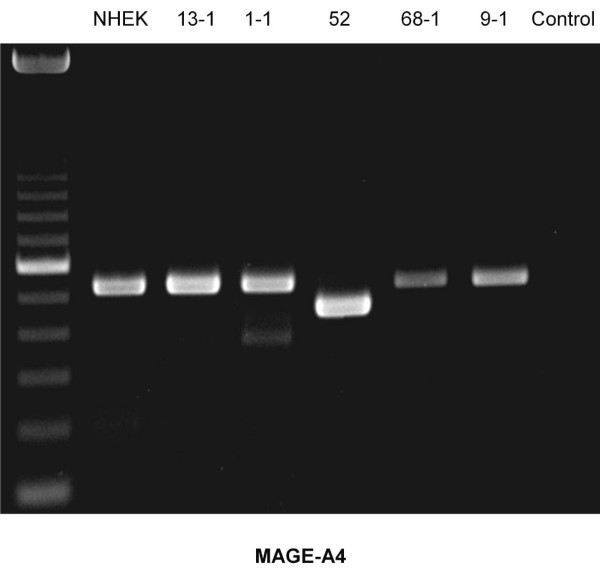
**RT-PCR blot of MAGE-A4 expression in cell lines examined**. Cell line PCI 52 has a splice variant. Besides this finding, there is a homogenous expression pattern. (left column = 100 bp marker, right column (control) = water).

**Figure 4:**
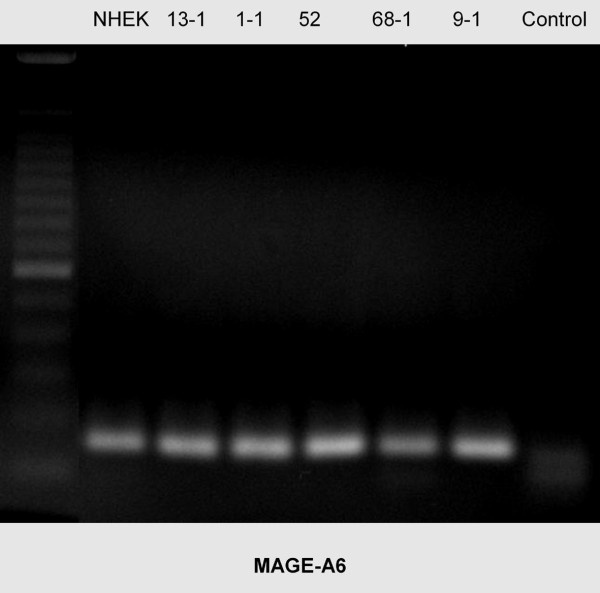
**RT-PCR blot of MAGE-A6 expression in the cell lines examined**. There is a homogenous expression pattern. (left column = 100 bp marker, right column (control) = water).

**Figure 5:**
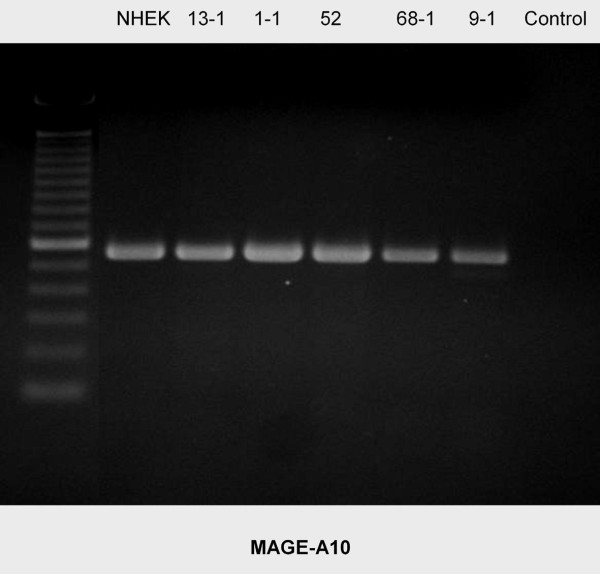
**RT-PCR blot of MAGE-A10 expression in the cell lines examined**. There is a homogenous expression pattern. (left column = 100 bp marker, right column (control) = water).

Using rt-PCR, MAGE-A antigens were detected in all cell lines examined (Figures 1, 2, 3, 4, 5). MAGE-A10 was also detected in all cell lines. This is in contrast to qrt-PCR which did not reveal any significant expression compared with the reference cell line. This can be explained by the high number of PCR-cycles (35). Even minimal amounts are amplified to sufficient portion for detection.

Some splicing variants of MAGE-A2 and MAGE-A4 were found. They can be seen in figure 1 (MAGE-A2) for the cell lines PCI 13-1, PCI 1-1, PCI 52 und PCI 9-1 and in figure 3 for the cell line PCI 52. Those splicing variants have been described before . The amplification of each of those variants is very strong compared with its counterpart (wild type). This should cause no bias regarding the amplification in qrt-PCR.

**Figure 6:**
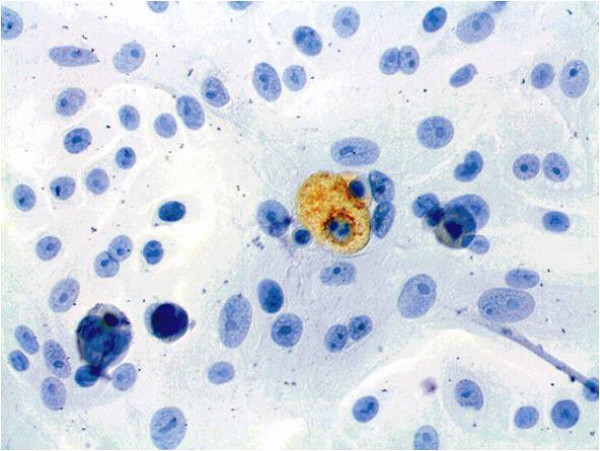
**Slide with NHEK cells stained immunocytochemically for MAGE-A and counterstained with hematoxylin (400× magnification)**. Only a single cell was found with antibody staining.

**Figure 7:**
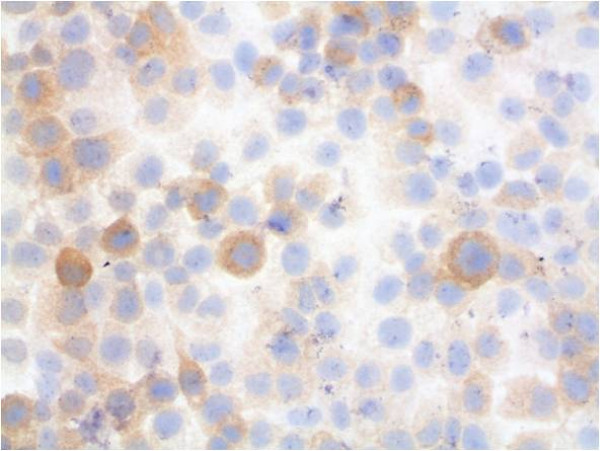
**Tumour cell line PCI 13-1 with immunocytochemical MAGE-A staining and hematoxylin counterstaining (400× magnification)**. Most cells are stained by the MAGE-A antibody. Some of the cells show a strong cytosolic staining.

**Figure 8:**
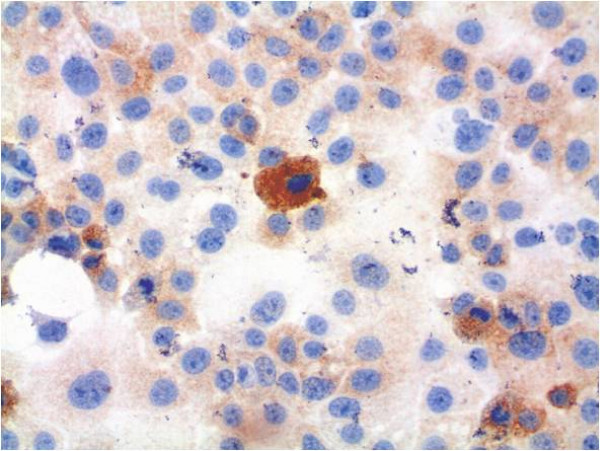
**Tumour cell line PCI 1-1 with immunocytochemical MAGE-A staining and simultaneous hematoxylin counterstaining (400× magnification)**. There is ubiquitous cytosolic staining, and some strong cytosolic staining by the MAGE-A Ab.

**Figure 9:**
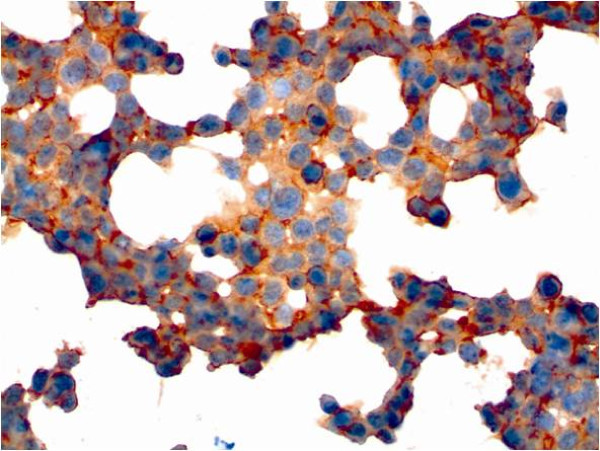
**Tumour cell line PCI 52 with immunocytochemical MAGE-A staining and simultaneous hematoxylin counterstaining (400× magnification)**. There is a ubiquitous cytosolic staining with simultaneous strong staining of the cell membranes.

**Figure 10:**
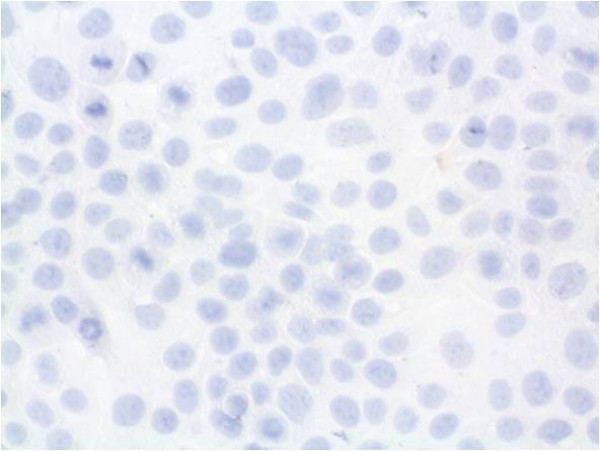
**Tumour cell line PCI 68-1 with immunocytochemical MAGE-A staining and simultaneous hematoxylin counterstaining (400× magnification)**. No staining with MAGE-A Ab of this tumour cell line was observed.

**Figure 11:**
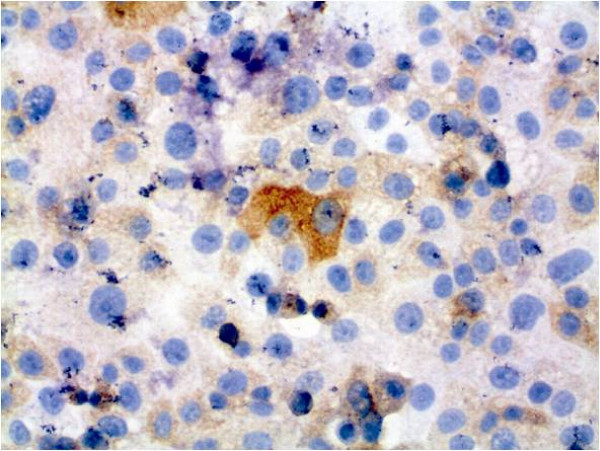
**Tumour cell line PCI 9-1 with immunocytochemical MAGE-A staining and simultaneous hematoxylin counterstaining (400× magnification)**. There is some weak cytosolic staining with sporadic stronger cytosolic staining with MAGE-A Ab.

For further analysis, immunocytochemical staining and hematoxylin counterstaining of cultured cells was performed (Figures 6, 7, 8, 9, 10, 11). The staining patterns differed significantly. In the NHEK adult keratinocyte cell line, only a sole cell in the whole slide was stained with the 57B antibody (Figure 6). The results for each cell line are described in detail.

### PCI 13-1

In rt-PCR all MAGE-A antigens could be amplified. This cell line showed a splicing variant for MAGE-A2. In qrt-PCR these cells expressed like cell line PCI 9-1, MAGE-A antigens -A2, -A3 and -A6 significantly. The expression levels were also comparable to cell line PCI 9-1. MAGE-A2 showed an expression of 11.46 a.u., MAGE-A3 9.94 a.u. (the highest value measured of the cell lines) and MAGE-A6 had an expression of 62.79 a.u.

The staining revealed a relatively equal pattern with some additional strong (about 15% of the cells) staining (Figure 7). The staining was mainly localised within the cytosol. The staining rate of the tumour cells was about 100%. This correlates with a multi-MAGE-A expression profile, with MAGE-A2, -A3, -A6 very high and MAGE-A4 being significantly weaker.

### PCI 1-1

In rt-PCR all MAGE-A antigens could be amplified. This cell line showed also a splicing variant for MAGE-A2. PCI 1-1 also has a similar multi-MAGE-A expression profile in qrt-PCR compared to PCI 13-1. This cell line expresses 3 different MAGE-A antigens (MAGE-A2, MAGE-A3 and MAGE-A6). MAGE-A2 and MAGE-A6 were expressed at a moderate level (MAGE-A2: 9.21 a.u.; MAGE-A3: 9.76 a.u.) and MAGE-A6 was expressed at a high level (64.54 a.u.). A MAGE-A4 expression was absent.

The cells of this tumour cell line were stained by mAb 57B in a very similar pattern compared to PCI 13-1 (Figure 8). Staining was also mainly localised within the cytosol and the staining rate was also approximately 100%. A strong staining was seen in about 10% of the tumour cells.

### PCI 52

The rt-PCR all MAGE-A antigens could be amplified and PCI 52 had also a splicing variant for MAGE-A2 and an additional splicing variant for MAGE-A4.

In qrt-PCR the expression profile of this cell line showed the most increased MAGE-A antigens. MAGE-A2, MAGE-A3, MAGE-A4, and MAGE-A6 were increased. PCI 52 is also the only cell line examined that showed a significant expression of MAGE-A4 in rt-PCR as well as in qrt-PCR (15.96 a.u.). This is remarkable because it was the only cell line with a MAGE-A4 splicing variant. The expression of MAGE-A2 was the highest of the cell lines examined and reached 15.39 a.u. Expression of MAGE-A6 was at the same level and reached 18.31 a.u. The value of MAGE-A3 expression was the lowest, at 3.49 a.u.

In this cell line, a very strong staining was seen. All cells showed a strong cytosolic and sometimes an even stronger cell membrane staining with mAb 57B (Figure 9). The staining rate was 100%.

### PCI 68-1

Despite the amplification of all MAGE-A antigens in rt-PCR PCI 68-1 only MAGE-A3 was significantly increased in qrt-PCR. But an expression of 2.97 a.u. was weak. In rt-PCR this cell line was the only one with the same splicing variant of MAGE-A2 compared with the reference cell line NHEK.

Within these tumour cells, no significant staining could be achieved by mAb 57B (Figure 10). The staining rate was about 0%. Only a very homogenous Hematoxylin staining was possible.

### PCI 9-1

In rt-PCR all MAGE-A antigens could be amplified. This cell line showed the same splicing variant for MAGE-A2 like PCI 13-1, PCI1-1 and PCI 52.

This cell line also expresses a group of MAGE-A antigens. As with PCI 1-1, this cell line highly expresses MAGE-A2, MAGE-A3 and MAGE-A6 in qrt-PCR. MAGE-A2 showed an expression of 10.67 a.u., MAGE-A3 of 8.88 a.u. and MAGE-A6 of 85.86 a.u. (the highest value measured of the cell lines examined).

In this cell line, there was a significant staining by mAb 57B (Figure 11). Nearly all of the cells were stained in the cytosol. The staining rate was about 100%. About 15% of the cells showed a stronger staining by mAb 57B.

The single qrt-PCR measurements listed in Table 2 were averaged for more a concise analysis.

**Table 2:** The table depicts the quantitative expression of the examined MAGE-A antigens in relation to the reference cell line of the adult keratinocytes (NHEK).

**MAGE**		**NHEK**	**13-1**	**68-1**	**1-1**	**52**	**9-1**
							

**A2**		1,00	12,18	1,67	11,74	4,24	14,46
		
		1,00	13,49	0,07	9,22	3,33	10,68
		
		1,00	8,71	1,10	6,67	2,00	6,86
		
	**M**	**1,00**	**11,46**	**0,95**	**9,21**	**15,39**	**10,67**

							

**A3**		1,00	14,90	3,46	15,97	5,90	16,80
		
		1,00	7,30	2,21	6,60	1,90	4,60
		
		1,00	7,62	3,24	6,70	2,66	5,23
		
	**M**	**1,00**	**9,94**	**2,97**	**9,76**	**3,49**	**8,88**

							

**A4**		1,00	2,09	0,93	0,51	20,07	0,19
		
		1,00	1,30	1,41	0,96	1,64	0,65
		
		1,00	1,34	0,83	0,46	26,17	0,29
		
	**M**	**1,00**	**1,58**	**1,06**	**0,65**	**15,96**	**0,37**

							

**A6**		1,00	98,53	2,43	114,00	38,93	197,00
		
		1,00	47,68	0,92	38,89	9,59	29,95
		
		1,00	42,15	0,16	40,73	6,40	30,62
		
	**M**	**1,00**	**62,79**	**1,17**	**64,54**	**18,31**	**85,86**

							

**A10**		1,00	0,05	0,38	0,29	0,02	0,05
		
		1,00	0,223	0,382	0,292	0,024	0,05
		
		1,00	0,173	1,093	0,278	0,091	0,337
		
	**M**	**1,00**	**0,15**	**0,62**	**0,29**	**0,05**	**0,15**

(M = mean)

## Discussion

The results are in accordance with reports in literature on the multiple expression of MAGE-A antigens in tumour cells that were harvested from primary solid cancers[10,12]. It was also possible to amplify MAGE-A antigens from the adult keratinocyte cell line NHEK in very low levels compared to the tumour cell lines. This might depend on the amplification cycles that facilitate the detection of very small amounts of those antigens. In immuncytochemistry those antigens were not detectable in NHEK. This corresponds with own results of immunohistochemical stainings in benign lesions of the oral mucosa, that did not show any MAGE-A antigens (data not shown). Among the five cell lines, only one expressed a single MAGE-A antigen. Three cell lines expressed 3 antigens, one cell line (PCI 13-1) showed an additional insignificant expression of a fourth antigen (MAGE-A4, 1.58 a.u.), and one cell line expressed 4 MAGE-A antigens. This proves that simultaneous heterogeneous expression of MAGE-A antigens is the rule and not the exception. The antigens show different expression patterns. Up until now, knowledge about the function of single MAGE-A gene products has been very limited[14]. Therefore, it is necessary to assess their function in regards to the course of disease and the prognosis. With this study on hand, further testing (e.g. for apoptosis or mitosis) in the examined cell lines in correlation with the expression of specific MAGE-A antigens is feasible. In the literature, there have been some remarks on the negative and positive influences of MAGE-A antigens on the course of cancer. The MAGE-A2 and MAGE-A6 gene products have been reported to bind to p53 (MAGE-A2 and MAGE-A6) and p73 (MAGE-A2) and to impair their function. This causes increased cell growth and decreased apoptosis[15,16] and leads to an increased resistance against chemotherapeutics (e.g. taxanes)[17,18]. These antigens were the most highly expressed antigens in the cell lines examined. They were found at significant levels in all tumour cell lines except PCI 68-1, and did not correspond to a T-(T2–T4) or N-stage (N0–N3) of the primary tumour tissue. Despite these criteria, the PCI 68-1 primary tumour tissue had the most differentiated cells (G1). Perhaps this might correlate with the missing expression of the MAGE-A antigens, but this has to be investigated in further studies. The expression of MAGE-A antigens depends on the demethylation of the promoter region[19]. Demethylation might be correlated with a higher degree of cell de-differentiation (G2 or G3)[19].

A higher expression of MAGE-A3 reduces the responsiveness of tumour cells to doxorubicin[17]. This antigen was expressed with at least borderline values in all tumour cell lines examined. Although doxorubicin is not a first-line antineoplastic agent for head and neck squamous cell carcinomas, these findings might be worthy of further investigations, as this could be one explanation, among others (e.g. sufficient levels of interferon-inducible protein IFI16)[20], for reduced responsiveness to doxorubicin.

A contradictory result is achieved by an elevated expression of MAGE-A4. Its gene product binds to the tumour protein gankyrin, which has a destabilising effect on retinoblastoma protein (pRb). Gankyrin binding to pRb results in its hyperphosphorylation, release of the E2F transcription factors, activation of DNA synthesis genes and unscheduled entry into the cell cycle[21,22]. Furthermore, it inhibits apoptosis by degradation of p53[23]. Binding of the MAGE-A4 gene product to gankyrin pRb is not destabilized, and degradation of p53 is reduced, The consequence of which is suppression of adhesion-independent tumour cell growth and formation of tumour cell clustering[21-24]. In our analyses, MAGE-A4 was only significantly expressed in tumour cell line PCI 52 (pT2N0M0G2). It was co-expressed with MAGE-A2, -A3 and -A6. This offers the opportunity to study the different behaviour of this tumour cell line as compared to other tumour cell lines expressing only MAGE-A2, -A3 and -A6. In these examinations, further determination of the role of MAGE-A4 in the progression and prognosis of oral squamous cell carcinoma might be possible.

In contrast to other investigators[10], no increased expression of MAGE-A10 was found in the tumour cell lines examined. This finding has to be verified in further studies. This is underlined especially in regard to an elevated expression of MAGE-A10 in a previous study of the authors in fetal keratinocytes[25].

In conclusion, oral and pharyngeal squamous cell carcinoma cell lines were found to express MAGE-A antigens. The MAGE-A antigens are expressed in groups of different antigens. This finding is supported by the literature[10,12]. The physiological functions of the MAGE-A gene products in cell differentiation and their possible implications on the course of cancerous disease and its prognosis are still unknown[9]. With the values presented in this study, further examinations regarding the functions of the MAGE antigens are possible. Furthermore, the evidence of these antigens makes them still interesting as possible targets for immunotherapy.

## Competing interests

The authors declare that they have no competing interests.

## Authors' contributions

UMR: study design, drafting of manuscript; AD: laboratory studies; TR: manuscript revision; JK: manuscript revision; TR: manuscript revision; OD: study design, manuscript revision

## Acknowledgements

The authors would like to thank Dr. Michael Kochel and Professor Alexander C Kübler for their assistance revising the manuscript.
